# Dosimetric Analysis of Axillary Lymph Node Coverage Using High Tangents in the Prone Position for Left-Sided Breast Cancers

**DOI:** 10.7759/cureus.23613

**Published:** 2022-03-29

**Authors:** Timothy D Malouff, Laura A Vallow, Wilza L Magalhaes, Danushka S Seneviratne, Mark R Waddle, Katherine S Tzou

**Affiliations:** 1 Department of Radiation Oncology, Mayo Clinic, Jacksonville, USA; 2 Department of Radiation Oncology, Mayo Clinic, Rochester, USA

**Keywords:** prone, micrometastatic, radiation, high tangents, breast

## Abstract

Aim/Objective: Prone positioning is often used to reduce the dose to organs at risk during adjuvant breast irradiation. High tangents are used with supine treatments in patients with the low-volume nodal disease to increase nodal coverage while minimizing toxicities. Our study aims to evaluate nodal coverage for patients treated in the prone position with high tangents.

Materials and Methods: Our study analyzed the plans for 20 patients with early-stage, left-sided breast cancers treated at our institution from 2018 to 2019. All patients were treated in the prone position. Axillary nodal levels I-III were contoured, and treatment plans were generated using high tangents. The heart, bilateral lungs, and breast tissue were retrospectively contoured. All plans were evaluated to a dose of 42.4 Gy in 16 fractions.

Results: Level I lymph node levels had a mean coverage of 99% of the prescription dose (range: 98-100%). Similarly, level II coverage was approximately 88% (range: 65-100%). The mean coverage for level III was approximately 25% (range: 0-52%). The mean heart dose, mean lung volume receiving ≥20 Gy (V20) for the bilateral lungs, and ipsilateral V20 were 1.69 Gy, 1.64%, and 3.56%, respectively.

Conclusion:Treating patients in the prone position with high tangents provides excellent coverage of axillary levels I and II, although there is minimal coverage of axillary level III. Prospective trials are needed to evaluate the clinical outcomes when treating patients with high tangents in the prone position.

## Introduction

Breast-conserving surgery followed by adjuvant radiation therapy has allowed a woman with early-stage breast cancer to achieve excellent locoregional control and overall survival while maintaining an acceptable level of cosmesis [1]. Additionally, sentinel lymph node biopsy (SLNB) has become a standard of care in early-stage breast cancer to more accurately evaluate for nodal metastases, although, until recently, the proper management for limited sentinel lymph node metastatic burden was controversial.

The use of high tangents has also been investigated for the use of low-volume nodal disease. Although not directly evaluating high tangents, the ACSOG Z0011 (Alliance) trial randomized patients to SLNB alone or axillary dissection, with SLNB resulting in noninferior survival [2]. In this study, approximately 50% of patients in both arms received high tangents, which may contribute to the noninferior outcomes in the SLNB cohort [3]. The European Organisation for Research and Treatment of Cancer (EORTC) 10981-22023 AMAROS trial took this concept a step further, and although it did not evaluate the use of high tangents, it supports the idea that radiation alone in the setting of a positive sentinel lymph node is sufficient [4]. Given the findings from these randomized studies, there is interest in using high tangents in the setting of limited lymph node disease burden identified on SLNB, as this technique intentionally targets the axillary lymph nodes while sparing the large field irradiation of regional nodal irradiation [5].

As adjuvant radiation therapy following breast conservation surgery has become increasingly used, there has been significant interest in decreasing the toxicities associated with treatment. To meet this goal, treatment in the prone position has been used in the adjuvant setting to provide adequate coverage while attempting to spare the lungs and heart. For example, previous dosimetric studies have shown that there is a reduced lung dose when treated in the prone position [6-11].

There is currently a lack of published data regarding combining high tangents and treating patients in the prone position for early-stage breast cancer patients with positive SLNB. Our study aims to fill the gaps in the literature by evaluating axillary nodal coverage and organ at risk (OAR) doses when treating patients in the prone position with high tangents.

## Materials and methods

To determine the nodal coverage when treating with high tangents in the prone position, a total of 20 patients treated at our institution for early-stage, left-sided breast cancers from 2018 to 2019 were identified. As our study was retrospective (all patients had completed treatment prior to the study and involvement in the study did not impact treatment delivery), our study did not require formal Institutional Review Board approval.

Only patients with left-sided breast cancer were included, as this would allow for an upper-end estimate of the average heart doses. All patients received adjuvant radiation therapy following lumpectomy and were planned in the prone position using standard tangents and field-in-field techniques as per our institutional standard for treatment. The CT simulation images were obtained, and a dosimetric analysis was performed retrospectively using reconstructed treatment plans with high tangents to evaluate nodal coverage.

**Figure 1:**
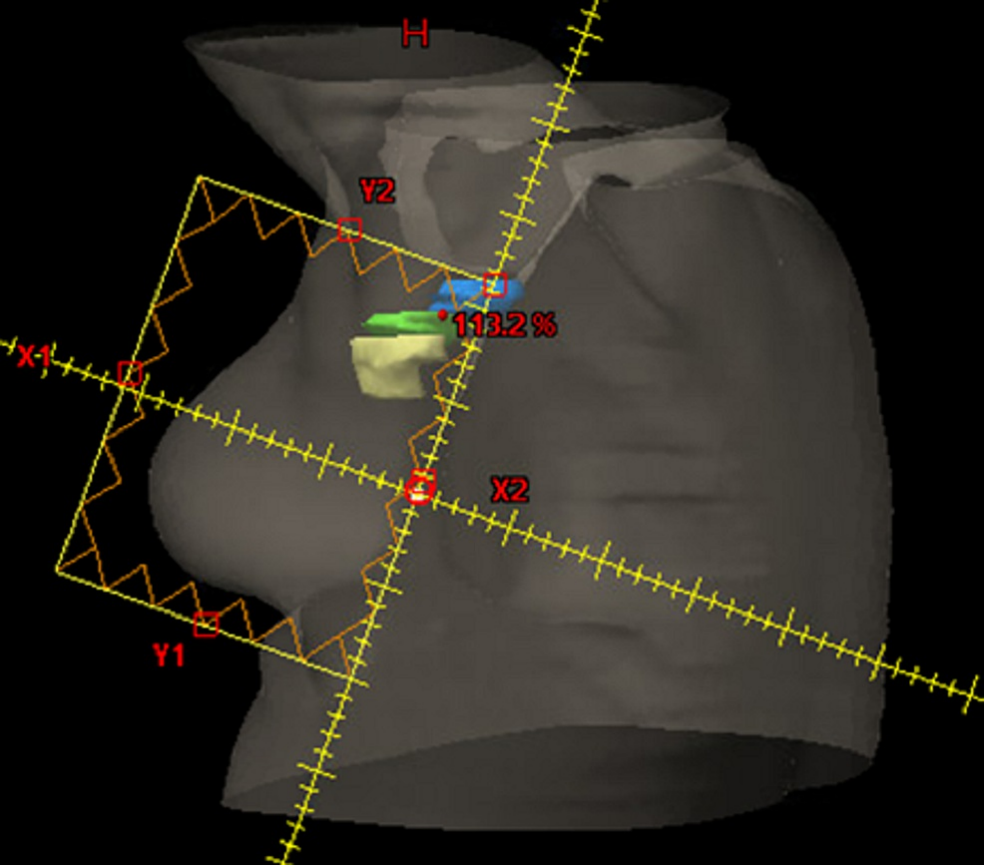
Representative beams-eye view of the lateral tangent for a patient treated for left-sided breast cancer. The superior border is the inferior aspect of the humeral head, with an inferior border approximately 2 cm below the inframammary line. Lymph node levels I (yellow), II (green), and III (blue) are contoured.

**Figure 2:**
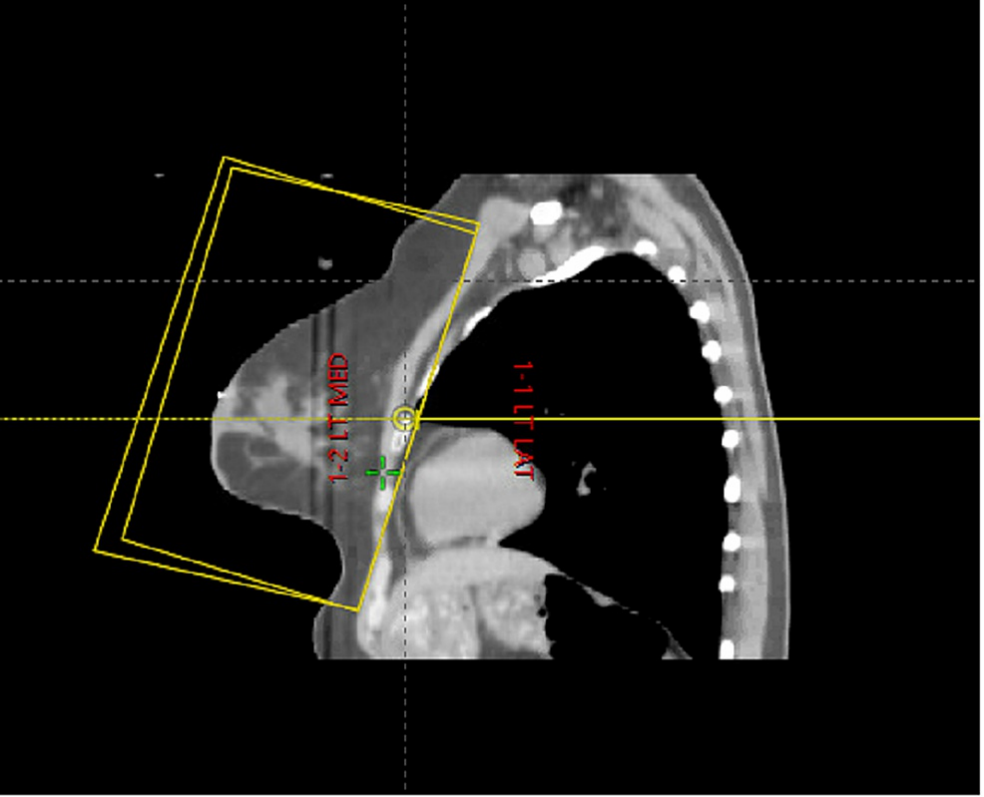
Representative CT simulation image in the sagittal plane of the field borders for high tangents.

**Figure 3:**
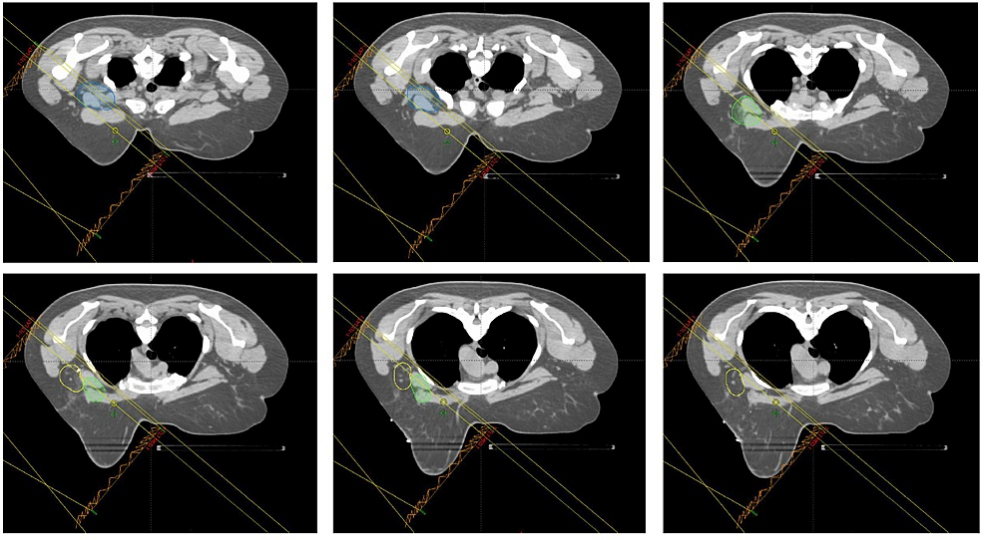
Representative CT simulation images in the axial plane from the superior-most border (left top) to the inferior-most border (bottom right). Lymph node levels I (yellow), II (green), and III (blue) are contoured and the field borders are included.

All patients at our institution have field borders marked with wire at the time of CT simulation and verified on the CT image set. Although field borders are initially based on bony anatomic landmarks, verification and any necessary modifications to the field borders are performed at the physician’s discretion at both the times of simulation and plan review to ensure adequate target coverage of the lumpectomy site or other high-risk disease areas. For all patients receiving standard tangents, the superior border is defined as the inferior aspect of the clavicle. This is expanded superiorly to the inferior aspect of the humeral head for high tangents (Figures 1-3).

The medial border is defined as the mid-sternum, and the lateral border is defined clinically as the mid-axillary line. The inferior border is defined as 2 cm below the inframammary line. Medial and lateral tangents are created with gantry and collimator rotations individualized to maximize target coverage while minimizing dose to the adjacent lung. Blocking and “field-in-field” techniques were performed to maximize coverage to the breast and limit OAR constraints as per our standard practice. No modifications to the high-tangent borders or blocking were made to specifically improve nodal coverage.

Axillary nodal levels I, II, and III were retrospectively contoured on each patient as per the Radiation Therapy Oncology Group (RTOG) breast cancer atlas [12,13]. The contours for each patient were created and independently reviewed by two physicians (TM and LV). Additionally, the heart, bilateral lungs, and breast tissue were retrospectively contoured for each patient for consistency. Treatment plans were developed using medial and lateral tangents to a dose of 42.4 Gy in 16 fractions. A representative beams-eye view of the field borders is shown in Figure 1. Nodal level coverage, ipsilateral lung volume receiving ≥20 Gy (V20), total lung V20, and mean heart dose were collected and analyzed using descriptive statistics.

## Results

Our study aimed to determine if nodal coverage when using high tangents in the prone position was adequate. There was excellent coverage of the axillary level I, with the mean coverage of 99% of the volume receiving prescription dose (standard deviation 1, range: 98-100%). In all, 14 of the 20 patients (70%) achieved 100% coverage of axillary level I, and 100% of patients achieved at least 95% coverage of axillary level I, suggesting sufficient coverage (Table 1). Similarly, the mean volume receiving the prescription dose for axillary level II was approximately 88% (standard deviation 11, range: 65-100). For axillary level II, one patient had 100% coverage, while seven patients (35%) had at least 95% coverage (Table 1). The coverage of axillary level III was less than the previous levels, with a mean coverage for axillary level III of approximately 25% of the volume receiving prescription dose (standard deviation 18, range: 0-52%; Table 1). When analyzing the primary treatment target of the ipsilateral breast, treatment with high tangents provided excellent coverage, with a mean coverage of 99.3% of the prescription dose (standard deviation 0.67, range: 96.8-100%).

**Table 1:** Mean coverage of lymph node levels receiving the prescription dose. In example, on average, 99% of axillary level I received the prescription dose.

Lymph node Level	Coverage	Standard Deviation (Range of coverage)
Level I	99%	1 (98-100% coverage)
Level II	88%	11 (65-100% coverage)
Level III	25%	18 (0-52% coverage)

Minimizing the dose to OARs, while providing excellent nodal coverage, is a primary aim of using high tangents. In regards to OARs, the mean heart dose for all patients was approximately 1.69 Gy (standard deviation 1.02 Gy, range: 0.56-4 Gy). The mean ipsilateral lung V20 was 3.45% (standard deviation 1.72, range: 0.8-7.7%). The mean total lung V20 was 1.64% (standard deviation 0.8, range: 0.4-3.7%; Table 2).

**Table 2:** Mean dose to relevant organs at risk. V20: lung volume receiving ≥20 Gy

Organ at risk constraint	Dose	Standard deviation	Range
Mean heart dose	1.69 Gy	1.02	0.56-4 Gy
Ipsilateral lung V20	3.45%	1.72	0.8-7.7%
Total lung V20	1.64%	0.8	0.4-3.7%

## Discussion

Our study suggests that high tangents can be used in patients requiring axillary coverage, such as those with a positive sentinel lymph node with no further axillary dissection while maintaining the dosimetric and logistical benefits of treatment in the prone position. In our study, there was adequate coverage of axillary levels I and II (mean coverage of 99% and 88%, respectively), although axillary level III had inadequate coverage, without significant detriment to the OARs.

Although there is currently a lack of studies investigating the use of high tangents in the prone position, the excellent coverage of axillary lymph node levels with high tangents in our study is in contrast to patients treated in the prone position with standard tangents, where multiple studies have reported on poor coverage of all axillary lymph node levels. Alonso-Basanta et al. reported on the mean dose to the axillary nodal stations when treating with standard tangents to a dose of 50 Gy. When in the prone position, axillary level I had a mean dose of approximately 11 Gy, axillary level II had 12.2 Gy, and level III had 8.2 Gy. Interestingly, the coverage was significantly lower for all axillary levels when treated in the prone position compared to the supine position [14]. Furthermore, a dosimetric study by Csenki et al. reviewed 100 breast cancer patients for axillary and internal mammary node coverage when treated in the prone or supine position with 3D conformal radiation to a dose of 50 Gy. As expected, patients in the prone position had lower rates of nodal coverage (V45 Gy of 3.0% for axillary level I) compared to the supine position. Notably, only 17% of patients received a mean dose of 45 Gy to axillary level I [15].

From this data, it is clear that patients treated with standard tangents in the supine position will not receive an adequate dose to the axillary nodes, and the expanded superior border for a high-tangent field is required to improve axillary coverage. For instance, in the study by Reznik et al., the authors found improved coverage with high tangents compared to standard tangents when treated in the supine position. In their study, axillary levels I and II had average coverage of 86% and 71%, respectively, with high tangents. Notably, axillary level III had mean coverage of approximately 73% of the prescribed dose, which is higher than the 25% reported in our study [16].

With these studies demonstrating increased axillary coverage when using high tangents in the supine position, there have been several small studies attempting to increase axillary nodal coverage in the prone position. Using the prone technique, MacDermed et al. reported subtherapeutic doses to the axillary lymph nodes in the prone position with standard tangents, with only 13% of axillary level I receiving 90% of the prescription dose, and 0% of axillary levels II to III receiving prescription dose when using standard tangents. The authors then analyzed 10 patients (5 right- and 5 left-sided cancers) with modified tangents to increase axillary coverage. All plans had 95% of axillary level I covered with at least 90% of the prescription dose, with a median of 65% of axillary level II covered with 90% of the dose [17]. Further, Shaikh et al. reviewed 41 patients treated in the prone position with high tangents and found the mean coverage of axillary level I to be approximately 89% and axillary level two to be 40%[18]. These findings are consistent with our study, where axillary levels I and II had 99% and 88% coverage, respectively, and suggest the dosimetric feasibility of treating with high tangents in the prone position. Although out of the scope of our study, further dosimetric analyses are required to determine if axillary coverage, especially axillary level II, and can be improved with modifications to the field borders or gantry angles to compensate for differences in anatomic positioning between supine and prone positions.

There is a lack of data regarding clinical outcomes when combining the prone position with high tangents, although previous studies have reported excellent clinical outcomes for SLNB-positive patients treated with high tangents in the supine position. For instance, in a study by Sanuki et al., 678 patients with early-stage breast cancer who underwent breast-conserving surgery and treatment with 2D or 3D planned high tangents were compared. Notably, the 5-year incidences of axillary failure in this group were 3.1% and 0.3% in the 2D and 3D groups, respectively, suggesting reasonable axillary control rates with high tangents [19]. Furthermore, Setton et al. reported 4-year local and regional control of 100% in 41 patients treated with breast-conserving surgery and were found to have a positive lymph node on SLNB without axillary lymph node dissection (ALND), comparable with comprehensive fields and standard tangents [20].

Although there are several other dosimetric and logistical benefits to treating patients in the prone position, the reduction of lung dose remains an important factor in deciding on treating in the prone position. To this end, lung dose remains a vital metric, especially when increasing the size of the treatment field. Similar to previous studies, our study also found very low lung doses with high tangents, although higher than those reported in the literature for patients treated with standard tangents. A systematic review found that the ipsilateral lung dose was lowest for patients treated in the prone position, with a mean dose of 1.2 Gy, compared to the supine position [21]. While the V20 to the ipsilateral lung is slightly higher in our data compared to patients treated with standard tangents, the absolute dose delivered to the ipsilateral lung remains low, likely resulting in a negligible increased risk for radiation pneumonitis [22]. Given the low absolute dose, the hypothesized improved locoregional control with better nodal coverage likely offsets the detriment of a higher lung dose, although this will have to be verified with clinical studies.

Importantly, we found no detriment in the mean dose delivered to the heart. Our data revealed a mean heart dose of approximately 1.69 Gy when using high tangents in the prone position, which is consistent with the mean heart dose reported in previous studies. For example, Alonso-Basanta et al. reported a mean heart dose of 1.5 Gy (standard deviation of 1.0 Gy) when treating patients in both the prone and supine position [14] when treated to a dose of 50 Gy using standard tangents. Similarly, the study by Yu et al. reported a mean heart dose of 1.19 Gy when treated in the prone position for all patients, increasing to 2.00 when accounting for only left-sided patients [7]. Although the study by Yu et al. used partial breast irradiation, the study demonstrates the increased heart dose when treating left-sided patients in the prone setting. Further investigation is needed to better characterize the dosimetric implications of high tangents on cardiac substructures, such as heart V5 and left anterior descending artery dose, as well as the clinical implications.

Although our data suggest the feasibility of using high tangents in the prone position when axillary nodal coverage is required, there are some limitations of our study inherent in dosimetric analyses that limit the current applicability of such techniques. First, although our data show sufficient dosimetric coverage with adequate OAR sparing, further clinical study is necessary to confirm equivalence in terms of oncologic outcomes and toxicity rates. Additionally, our study is limited to patients with left-sided breast cancer to determine the feasibility of combining the techniques in regards to heart dose, although more complete dosimetric analysis involving the bilateral breasts will be necessary prior to the widespread application of these techniques.

## Conclusions

Treating patients with early-stage, left-sided breast cancer with high tangents in the prone position provides excellent coverage of axillary levels I and II, although there is minimal coverage of axillary level III. Additionally, there was no compromise in the dose to the OARs when using high tangents. Based on these findings, treating patients with high tangents in the prone position can be considered for left-sided breast cancers when nodal coverage of levels I and II is required, such as in the setting of sentinel lymph node positivity, although further prospective trials are needed to evaluate the clinical outcomes and toxicities.
